# Hydroxycamptothecin Inhibits Peritendinous Adhesion *via* the Endoplasmic Reticulum Stress-Dependent Apoptosis

**DOI:** 10.3389/fphar.2019.00967

**Published:** 2019-09-04

**Authors:** Zhixiao Yao, Wei Wang, Jiexin Ning, Xiangqi Zhang, Wei Zheng, Yun Qian, Cunyi Fan

**Affiliations:** ^1^Department of Orthopaedics, Shanghai Jiao Tong University Affiliated Sixth People’s Hospital, Shanghai, China; ^2^Department of Plastics, Binzhou People’s Hospital, Binzhou, China; ^3^Department of Pharmacy, Shanghai Jiao Tong University Affiliated Sixth People’s Hospital, Shanghai, China

**Keywords:** hydroxycamptothecin, peritendinous fibrosis, TGF-β1, IRE1, ATF-6

## Abstract

Traumatic peritendinous fibrosis is a worldwide clinical problem resulting in severe limb disability. Hydroxycamptothecin (HCPT) is an anti-neoplastic drug widely exploited in clinical practice. It has shown potential of anti-fibrosis in recent years. We previously demonstrated that HCPT inhibited the characterization of fibrosis *in vitro*. However, it is still unclear whether it ameliorates peritendinous adhesion in an *in vivo* animal tendon injury model. The underlying mechanism is also worth investigating. The present study aims to determine whether HCPT inhibits tendon adhesion and to explore the underlying mechanisms. In a rat tendon injury model, we observed that topical application of HCPT significantly attenuated peritendinous adhesion as revealed by the results of macroscopic observation, biomechanical, histological, immunohistochemical evaluation, western blot, and quantitative PCR (q-PCR) analyses. Furthermore, western blot and q-PCR analyses revealed that this phenomenon is correlated with HCPT activation of endoplasmic reticulum (ER) stress. In addition, *in vitro* studies show that HCPT significantly inhibits fibroblast proliferation and induces apoptosis by reducing the expression of extracellular matrix (ECM) proteins COL3A1 and α-smooth muscle actin (α-SMA). Finally, we employed small interfering RNA (siRNA) to target inositol requiring kinase 1 (IRE1) and activated transcription factor 6 (ATF-6) to verify that the effect of inhibitory fibrosis of HCPT disappears after knockdown of ATF-6 and IRE1, thereby suggesting that an anti-fibrotic effect of HCPT is mediated by the ER-dependent apoptotic pathway. In conclusion, our results indicate that HCPT inhibits peritendinous fibrosis through the ER-dependent apoptotic pathway and might serve as a potential solution to prevent traumatic peritendinous adhesion.

## Introduction

The adhesion of injured tendons is currently one of the most frequent and severe complications that occur after tendon injury. Excessive proliferation of fibrous tissues between the tendon and the surrounding synovial sheath after injury compromises tendon excursion and eventually leads to limb dyskinesia, which is a complex clinical problem (Manske, 1988; Sharma and Maffulli, 2005; Docheva et al., 2015). Currently, a variety of strategies are employed to prevent peritendinous adhesions (Tang, 2005; Ishiyama et al., 2010; Ishiyama et al., 2011; Xia et al., 2012; Jiang et al., 2014; Zheng et al., 2018; Liu et al., 2019), but the efficiency of these approaches is suboptimal and mechanism of peritendinous tissue fibrosis has not been clarified. Therefore, an effective method to inhibit tendon adhesion at key locations of pathogenesis remains to be developed. Increasing evidence suggests that peritendinous fibrosis is characterized by excessive deposition of ECM components including collagen and α-SMA (Wynn and Ramalingam, 2012). It is believed that activated myofibroblasts are the main culprit in the deposition of ECM. Transforming growth factor (TGF)-β, abundantly expressed in fibrotic diseases, is currently considered to be a key mediator of these disorders (Ding et al., 2014; Sosulski et al., 2015; Musso et al., 2016; Cianfarani et al., 2018; Russo et al., 2019; Huynh and Chai, 2019). A growing body of research suggests that TGF-β1 is the most significant regulatory mediator of myofibroblasts. In other words, TGF-β1 stimulates the increase of α-SMA-positive differentiated myofibroblasts (Desmouliere et al., 1993). However, on the other hand, studies have shown that blocking TGF-β, to prevent peritendinous fibrosis, may be counterproductive. The latest research reports that TGF-β promotes up-regulating the expression of collagen during the tendon healing process, while destroying the ECM balance that leads to fibrosis. Herein, TGF-β exhibits multiple pleiotropic effects during tendon repair (Farhat et al., 2012).

Hydroxycamptothecin (HCPT), one of a series of Chinese native plant camptothecin, is a cell cycle-specific DNA topoisomerase I inhibitor. Current research indicates that HCPT has an inhibitory effect on the proliferation of various tumor cells with few side effects. Therefore, HCPT is widely used as an anti-tumor drug with a favorable foreground (Jaxel et al., 1989; Zhang et al., 1999; Garcia-Carbonero and Supko, 2002; Wu et al., 2009; Guo et al., 2018). Recently, HCPT was reported to reduce epidural fibrosis by inhibiting fibroblast hyperproliferation and inducing fibroblast apoptosis (Sun et al., 2008; Yang et al., 2011). Similarly, it has also been reported to prevent postoperative glaucoma scar adhesion by inducing apoptosis of human tendon capsule fibroblasts (Yin et al., 2013). Our previous study showed that HCPT may have beneficial effects on tendon adhesion by inducing apoptosis in human fibroblasts (Yao et al., 2019), however, the mechanism may require further investigation.

The ER is essential for the synthesis, folding, assembly, modification and transport of nascent proteins. When the protein fails to fold correctly, the ER activates a conserved adaptive cellular mechanism termed the unfolded protein response (UPR) (Harding et al., 1999). A short-term and mild UPR could restore protein folding homeostasis. However, if cells fail to recover from ER stress, apoptosis is triggered. ER stress is recognized to help regulate cell function and fate (Wang and Kaufman, 2016), thereby contributing to the pathological mechanisms of closely related diseases including inflammation, immunity, tumor, angiogenesis and neurodegenerative diseases (Grootjans et al., 2016; Cubillos-Ruiz et al., 2017; Cubillos-Ruiz et al., 2017; Hetz and Saxena, 2017). Prolonged stress or a failed adaptive response results in glucose-regulated protein 78 (GRP78/BiP) accumulation and activation of the downstream signaling molecule PRKR-like ER kinase (PERK), IRE1 and ATF-6. Activated PERK phosphorylation increases C/EBP homologous protein (CHOP/GADD153) expression and regulates Bax/Bcl-2 ratio, thereby triggering apoptosis (Gotoh et al., 2004; Roussel et al., 2013). Similarly, IRE1 phosphorylation can induce apoptosis by increasing CHOP expression. Recent work has confirmed that ATF-6 and IRE1 and their downstream molecules play important roles in ER-induced apoptosis (Senkal et al., 2011; Zhang et al., 2018; Chern et al., 2019; Zhang et al., 2019). In addition, HCPT was reported to induce fibroblast apoptosis through the PERK pathway *in vitro* (Yao et al., 2019). However, the entire mechanism remains to be further investigated.

The present study aims to determine whether HCPT could inhibit tendon adhesion through the IRE1 and/or ATF-6 signaling pathways. We tested our hypothesis *in vitro* by manipulating the cellular activity of IRE1 and ATF-6 using siRNA technology and *in vivo* in a rat tendon injury model. Our results indicate that HCPT might mediate fibroblast apoptosis *via* IRE1 and ATF-6 preventing tendon adhesion. It provides a novel and promising strategy for the prophylactic and therapeutic application of tendon adhesion.

## Materials and Methods

### Tendon Injury Model

Male Sprague-Dawley (SD) rats were purchased from the Shanghai Laboratory Animal Company (Shanghai, China) and were fed in a specific pathogen-free (SPF) environment. Animal welfare was offered to all the experimental animals and approved by the Animal Care Committee of Shanghai Jiao Tong University Affiliated Sixth People’s Hospital (No.2019-0239), and all procedures were conducted in accordance with standard guidelines. Adult SD rats (weighing 250 to 300 g) were randomly assigned to the control and HCPT treated groups (n = 20 per group). For the tendon injury surgery (Chen et al., 2017), rats were anesthetized by intraperitoneal injection of pentobarbital sodium (40 mg/kg). The left hind limb of each rat was sterilized with iodophor. A 2 cm “S” shaped incision was made on the skin to expose the thick and thin strand of the Achilles tendon. The thin layer of the Achilles tendon was removed, and a cut was made into the middle part of the thick stand of the Achilles tendon. The Achilles tendon was repaired with a 6-0 polypropylene suture (Ethicon, Edinburgh, UK). HCPT powder was dissolved in a dilute alkali solution and diluted with normal saline (9 mg/ml). Different concentrations (0.01, 0.05 and 0.1 mg/ml) of HCPT or normal saline (9 mg/ml) were applied to the repaired tendon site for 5 min (Sun et al., 2008; Yang et al., 2011) with a cotton pad (4 × 4 mm). After removing the cotton pad, the repaired tendon was washed with saline three times to remove the remaining HCPT. The skin was sutured at the surgical site with 4-0 silk. Animals were sacrificed 3 w after tendon injury and used for analysis.

The macroscopic evaluation of adhesion was performed by three independent investigators using an adhesion grading system (Jiang et al., 2014; Zheng et al., 2018). Hematoxylin-eosin (HE) staining and Masson staining were performed according to standard procedures and analyzed by light microscopy (Zheng et al., 2018). Histological evaluation was performed by three independent observers using a histological scoring system as previously described (Jiang et al., 2014; Zheng et al., 2018).

The maximum tensile strengths of five freshly collected samples in each group were evaluated using a biomechanical analyzer (Instron, Norwood, MA, USA) as previously described (Zheng et al., 2018) to assess tendon healing. Histologic tendon healing was scored as previously described (Jiang et al., 2014; Zheng et al., 2018).

### Hydroxyproline Content

The hydroxyproline (Hyp) content in the tendon tissue was measured according to standard protocols. First, collected tendon tissue was hydrolyzed in concentrated HCl. After 0.5 ml of chloramine-T reagent was added to the solution, the mixture was incubated for 20 min at 25°C and the solution was then incubated with 0.5 ml of Ehrlich reagent for 15 min at 65°C. The absorbance was measured at 550 nm.

### Cell Culture and Treatments

The rat fibroblast was skin-derived fibroblast cell line and was purchased from the Cell Bank of Type Culture Collection of the Chinese Academy of Sciences (Shanghai, China). The fibroblasts were cultured in DMEM supplemented with 10% fetal bovine serum (FBS) (Gibco, Carlsbad, CA, USA) and 1% penicillin/streptomycin (PS) (Gibco, Carlsbad, CA, USA), and cultured at 37°C in the 5% CO_2_ incubator. The cells were passaged at 80% confluence. The fibroblasts were treated with 2 ng/ml TGF-β1 (Minneapolis, MN, USA) and/or 1 μg/ml HCPT (Santa Cruz, CA, USA). SiRNAs targeting IRE1 and ATF-6 were purchased from GenePharma (Shanghai, China). Cells were transfected using Lipofectamine 2000 (Invitrogen, Carlsbad, CA, USA) according to the manufacturer’s instructions. Six h after transfection, the medium was replaced with normal fresh medium. The silencing efficiency of siRNA was assessed by western blot.

### Immunohistochemistry and Immunofluorescence

Immunohistochemical staining and immunofluorescence (IF) were performed with anti-COL3A1 and α-SMA antibodies (Abcam, Cambridge, MA, USA) according to standard protocols and analyzed under an optical microscope. Cells or tendon sections were incubated with the primary antibody overnight at 4°C. Cells or tendon sections were incubated with the secondary antibody (Abcam, Cambridge, MA, USA) for 30 min followed by washing three times with phosphate-buffered saline (PBS). For nuclear staining, cells or tendon sections were fixed in medium containing 4’,6-diamidino-2-phenylindole (DAPI) (Gibco, Carlsbad, CA, USA).

### Cell Proliferation Analysis

Cell viability was assessed using a Cell Counting Kit-8 kit (CCK-8; Dojindo, Kumamoto, Japan). Fibroblasts were cultured in 96-well plates for 24 h and then followed by TGF-β1 and/or HCPT treatment for 24 h. Then each well was co-cultured with CCK-8 reagents for 2 h at 37°C. The absorbance was measured at 450 nm.

Dead/live staining was performed using a Dead/Live staining kit (Invitrogen, Eugene, OR, USA) after TGF-β1 and/or HCPT treatment for 24 h. The red fluorescence indicated dead cells, while bright green fluorescence indicated live cells.

5-Ethynyl-2′-deoxyuridine (EdU) staining kit (Gibco, Carlsbad, CA, USA) was employed to assess cell proliferation. Fibroblasts were treated with TGF-β1 and/or HCPT for 24 h and co-cultured with an EdU working solution overnight. Cells were then fixed with 4% PFA solution and treated with 0.5% Triton X-100. Fibroblasts were incubated with Apollo reaction mixture and stained with Hoechst 33342 for 30 min. The results are expressed using the EdU positive nuclei analysis.

### Flow Cytometry Analysis

After TGF-β1 and/or HCPT treatment for 24 h, the fibroblasts were incubated with annexin V-fluorescein isothiocyanate (FITC) and propidium iodide (PI) working solution (BD Pharmingen, San Diego, CA, USA) for 30 min at room temperature. Analysis was performed using a flow cytometer (Beckman Coulter, Brea, CA, USA).

### Western Blot

Tensile tissue or treated fibroblasts were lysed in radioimmunoprecipitation buffer (RIPA) containing protease and phosphatase inhibitors as previously described (Jiang et al., 2014). A BCA Protein Assay Kit (Thermo Fisher Scientific, IL, USA) was employed to determine protein concentration. Twenty micrograms of lysate were loaded onto a 10 to 15% sodium dodecyl sulfate polyacrylamide gel (SDS-PAGE) for separation and then electrotransferred to a polyvinylidene fluoride membrane (PVDF; Millipore, Bedford, Massachusetts, USA). Membranes were incubated with primary including anti-COL3A1, anti-α-SMA, anti-ATF-6, anti-phospho-IRE1 (anti-p-IRE1), anti-CHOP, anti-Bcl-2 (Abcam, Cambridge, MA, USA), anti-GRP78 (glucose regulated protein 78), anti-Bax, and anti-β-actin (Cell Signaling Technology, Danvers, MA, USA) and secondary antibody (Abcam, Cambridge, MA, USA). β-Actin was used for standardization. A semi-quantitative analysis was performed using ImageJ software.

### Quantitative PCR

Total RNA was extracted from fibroblasts and tendon tissue using TRIzol reagent (Invitrogen, Carlsbad, CA, USA) as described previously (Chen et al., 2017). Primer sequences were shown in **Table 1**. GAPDH was used as a standardized internal reference.

**Table 1:** The Q-PCR primer sequences for rat.

Genes	Primer sequences	
α-SMA	Forward	CACCATCGGGAATGAACGCTTC
	Reverse	CTGTCAGCAATGCCTGGGTA
COL3A1	Forward	AGGTGGGTACACTGTAGCCT
	Reverse	GATCGCATAGGTGACAGGTGTT
GRP78	Forward	CTGGGTACATTTGATCTGACTGG
	Reverse	GCATCCTGGTGGCTTTCCAGCCATTC
CHOP	Forward	GTCTCTGCCTTTCGCCTTTG
	Reverse	CTACCCTCAGTCCCCTCCTC
Bax	Forward	TTTTCCTGGGATGAATGGGG
	Reverse	TGAGGTTTATTGGCACCTCC
Bcl-2	Forward	CAGCTGCACCTGACGCCCTT
	Reverse	CCCAGCCTCCGTTATTCTGGA
IRE1	Forward	TGGACGGACAGAATACACCA
	Reverse	TGGACACAAAGTGGGACATC
ATF-6	Forward	GGATTTGATGCCTTGGGAGTCAGAC
	Reverse	ATTTTTTTCTTTGGAGTCAGTCCAT
GAPDH	Forward	CACTGAGCATCTCCCTCACAA
	Reverse	TGGTATTCGAGAGAAGGGAGG

### Statistical Analysis

Data were showed as the mean ± the standard deviation (SD). Differences were analyzed using the paired Student’s t test and one-way ANOVA for multiple comparisons. Differences were considered to be statistically significant at *P* < 0.05.

## Results

### HCPT Suppresses Injury-Induced Tendon Adhesion *In Vivo*

**Figure 1:**
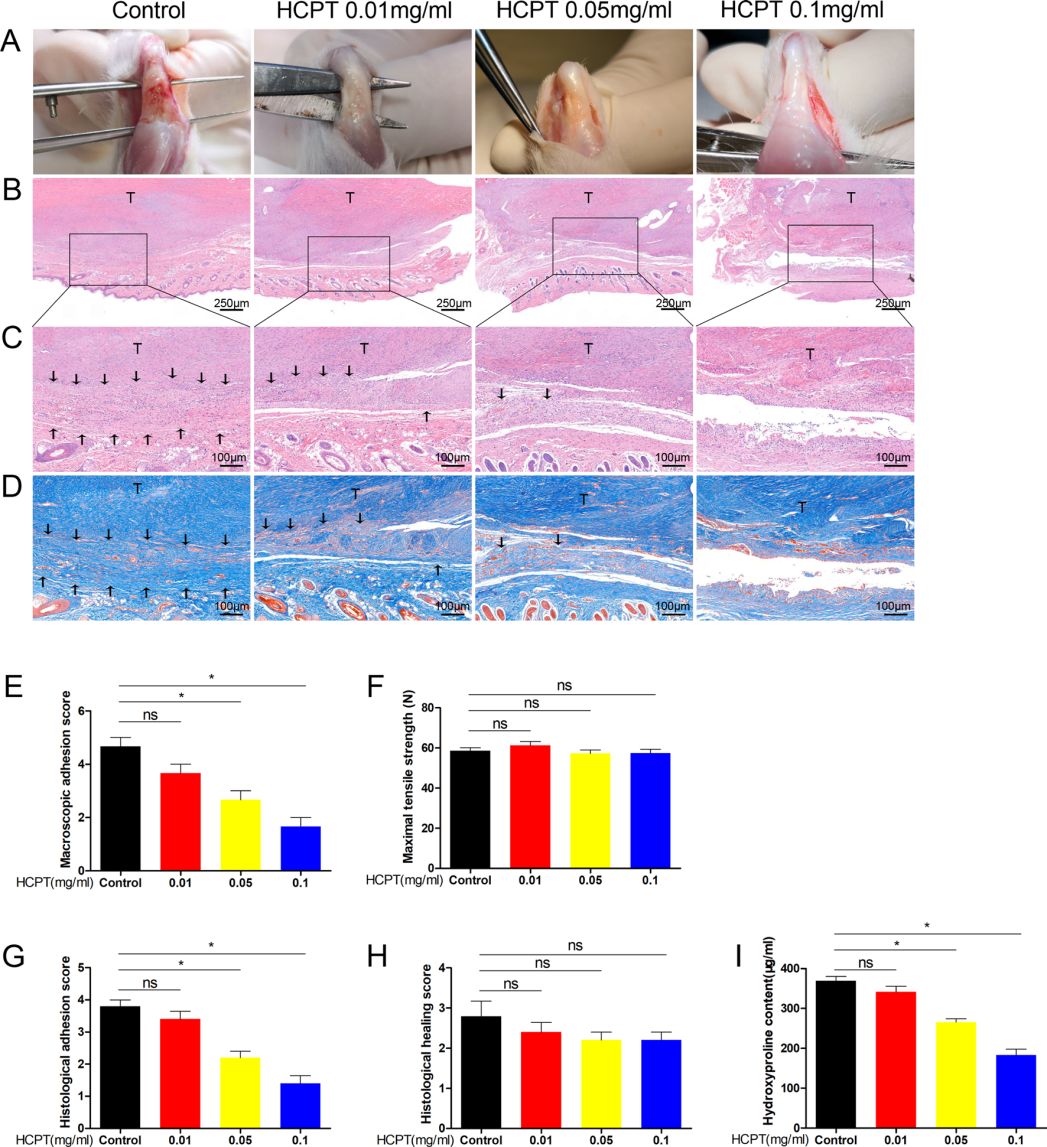
HCPT alleviated peritendinous adhesion in tenon-injured rats. Different concentrations of HCPT (0, 0.01, 0.05, and 0.1 mg/ml) were applied locally to the repaired tendon site for 5 min, and the tendons were examined 3 weeks later. **(A)** Macroscopic evaluation of peritendinous adhesions. **(B**-**D)** Histological assessment of tendon adhesions. **(E)** Macroscopic adhesion score. **(F)** Maximum tensile strength. **(G**, **H)** Histological score and tissue healing score. **(I)** Hydroxyproline content. Black arrow indicates adhesion tissue. T: tendon. Data are expressed as the mean ± SD of five independent samples. *P < 0.05. ns: not significant.

The tendons of the rats were damaged and then treated accordingly. All rats were euthanized 3 weeks after surgery and their tendons were tested for subsequent experiments. A group subjected to tendon injury without HCPT treatment was used as a control. Compared with that in the control group, the topical application of HCPT significantly reduced the degree of tendon adhesion in a concentration-dependent manner. Thick fibrotic tissues were found around the tendons of the control group, which could only be distinguished from the tendon through close anatomical examination. However, few tissues were found in the 0.05 mg/ml and 0.1 mg/ml HCPT groups (**Figure 1A**). HE staining revealed a significant reduction in hyperproliferative fibroblasts and inflammatory cells after HCPT treatment compared with their levels in the control group, with the 0.1 mg/ml treatment group having the most marked difference (**Figures 1B, C**). Masson staining showed a significant reduction in ECM deposition after HCPT treatment (**Figure 1D**).

Gross observations of peritendinous adhesions were performed, and the adhesion grade score of the 0.1 mg/ml HCPT treatment group was significantly less than those of the 0.01 mg/ml, 0.05 mg/ml and the control groups. However, there was no statistical difference between 0.01 mg/ml and the control group (**Figure 1E**). The histological scores were consistent with this result (**Figure 1G**). We further tested the content of hydroxyproline, which is the main component of collagen and reflects the degree of tendon adhesion. The data showed that the 0.1 mg/ml HCPT group had the lowest hydroxyproline content (**Figure 1I**). Altogether, these findings indicate that topical application of HCPT effectively reduces damage-induced tendon adhesion. In addition, it should be noted that no significant difference was observed between the groups in terms of maximum tensile strength and histological healing score (**Figures 1F, H**), suggesting that topical application of HCPT did not affect tendon healing.

**Figure 2:**
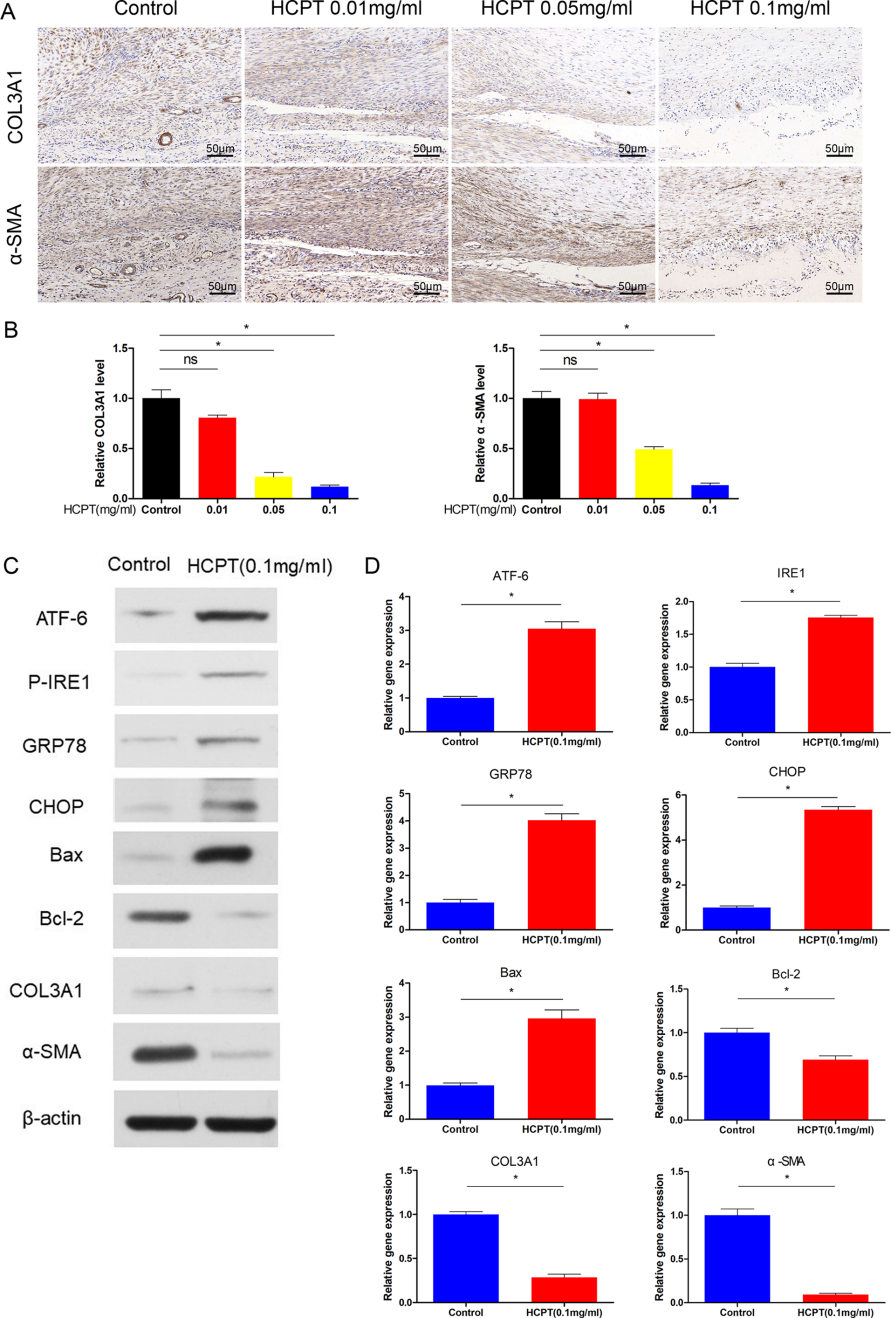
HCPT inhibited adhesion and activated ER stress *in vivo*. Different concentrations of HCPT (0, 0.01, 0.05, and 0.1 mg/ml) were applied locally to the tendon surgery site for 5 min, and the tendon was collected 3 weeks later. **(A)** Representative images of the immunohistochemical staining of COL3A1 and α-SMA. **(B)** Relative levels of COL3A1 and α-SMA. Tendons were treated with or without HCPT and collected 3 weeks after surgery for testing. **(C)** Representative immunoblot images of ATF-6, P-IRE1, GRP78, CHOP, Bax, Bal-2, COL3A1, and α-SMA. **(D)** Q-PCR analysis of the mRNA expression levels of ATF-6, IRE1, GRP78, CHOP, Bax, Bal-2, COL3A1 and α-SMA. Data are showed as the mean ± SD of five independent samples. *P < 0.05. ns: not significant.

To further assess the effect of HCPT on peritendinous adhesions *in vivo*, we used immunohistochemistry (IHC) to determine the expression of ECM. The data showed that HCPT treatment significantly reduced the expression of COL3A1 and α-SMA. Specifically, the 0.1 mg/ml HCPT treatment group had the lowest expression, but there was no statistical difference between the 0.01 mg/ml group and the control group (**Figures 2A, B**). This is consistent with the previous results and supports the protective effect of HCPT on peritendinous fibrosis.

### ER-Stress is Induced by HCPT *In Vivo*

Next, the protein and mRNA expression levels of ER stress genes were determined by western blot and q-PCR. The western blot results showed that ER stress proteins such as ATF-6, P-IRE1, GRP78, and CHOP were increased after treatment with 0.1 mg/ml HCPT 3 weeks after surgery. Furthermore, HCPT treatment significantly reduced the expression of Bcl-2 and increased the Bax/Bcl-2 ratio (**Figure 2C**). The q-PCR results support these conclusions (**Figure 2D**). In addition, western blot and q-PCR showed that the expression of COL3A1 and α-SMA was significantly decreased by 0.1 mg/ml HCPT treatment (**Figures 2C, D**). These data indicate that HCPT activates ER stress while decreasing ECM deposition.

### HCPT Inhibits Fibroblast Proliferation and Induces Apoptosis *In Vitro*

**Figure 3:**
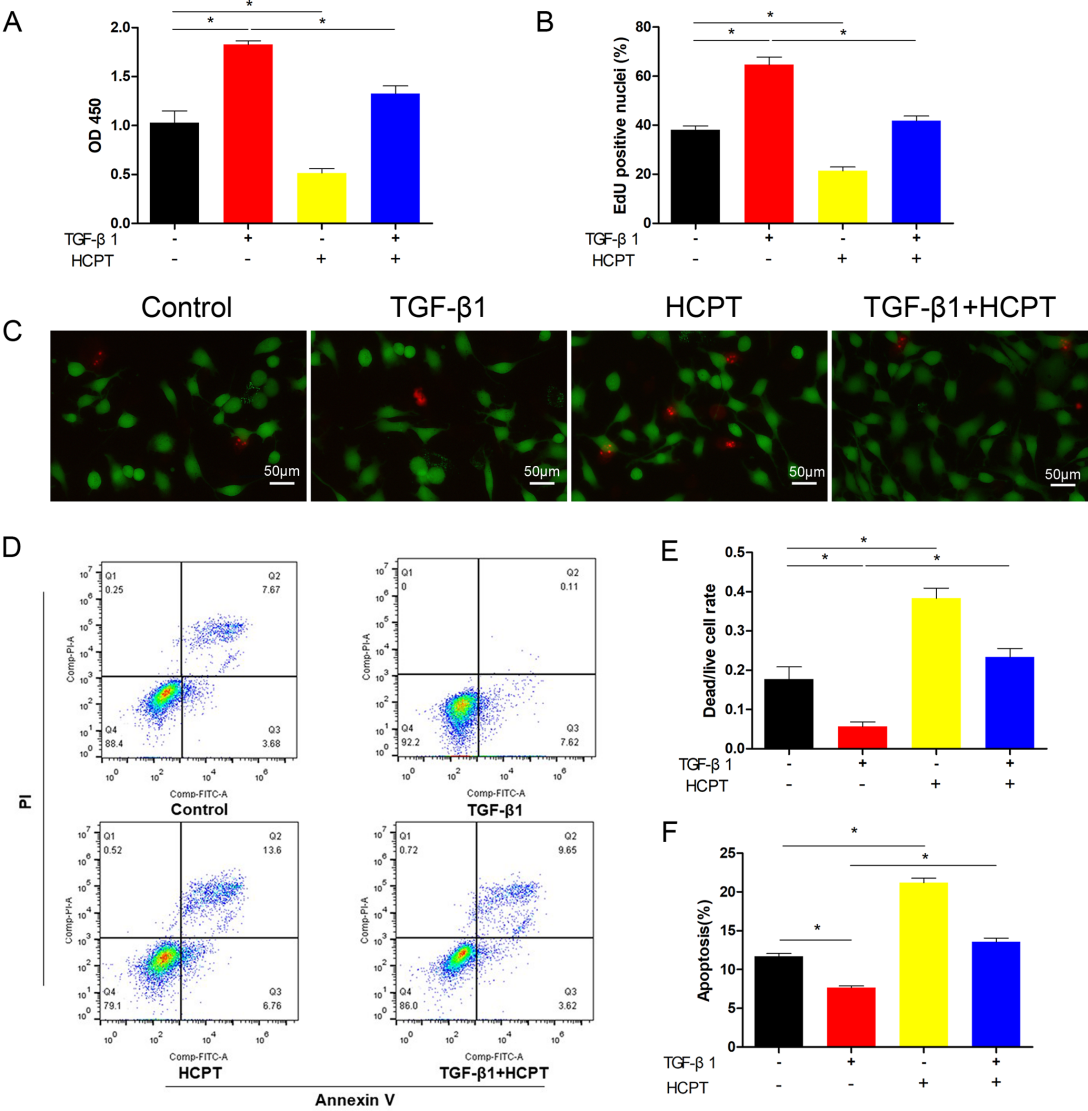
Effects of HCPT on the proliferation and apoptosis of fibroblasts. Fibroblasts were incubated with TGF-β1 (2 ng/ml) in the absence or presence of HCPT (1 μg/ml) for 24 h. **(A)** CCK-8 assay of cell viability. **(B)** Quantitative analysis of EdU staining. **(C)** Representative images of dead/live staining and **(E)** dead/live rate analysis. **(D)** Representative pictures of apoptotic flow cytometry and **(F)** quantitative analysis of fibroblasts apoptosis. Data are expressed as the mean ± SD of five independent samples. *P < 0.05.

Because of the role of TGF-β1 in fibrotic lesions, we evaluated cell proliferation and apoptosis by treating fibroblasts with TGF-β1 (2 ng/ml) and/or HCPT(1 μg/ml) for 24 h. The CCK-8 results indicated that HCPT alone reduces fibroblast activity and attenuates the increased cell activity by TGF-β1(**Figure 3A**). The EdU staining results showed that TGF-β1 induces fibroblast proliferation, whereas HCPT significantly inhibits this effect (**Figure 3B**). Dead/live kits were employed to measure the dead/live cell rate of the fibroblast. The results showed that with or without TGF-β1, HCPT increased the dead/live rate of fibroblasts, indicating that HCPT promotes fibroblast apoptosis (**Figures 3C, E**). Flow cytometry analysis showed that TGF-β1 reduces fibroblast apoptosis, but co-treatment with HCPT significantly promotes fibroblast apoptosis (**Figures 3D, F**). In summary, our data indicates that HCPT significantly inhibits proliferation and promotes apoptosis in fibroblasts.

### HCPT Attenuates TGF-β1-Induced Fibrosis *In Vitro*

**Figure 4:**
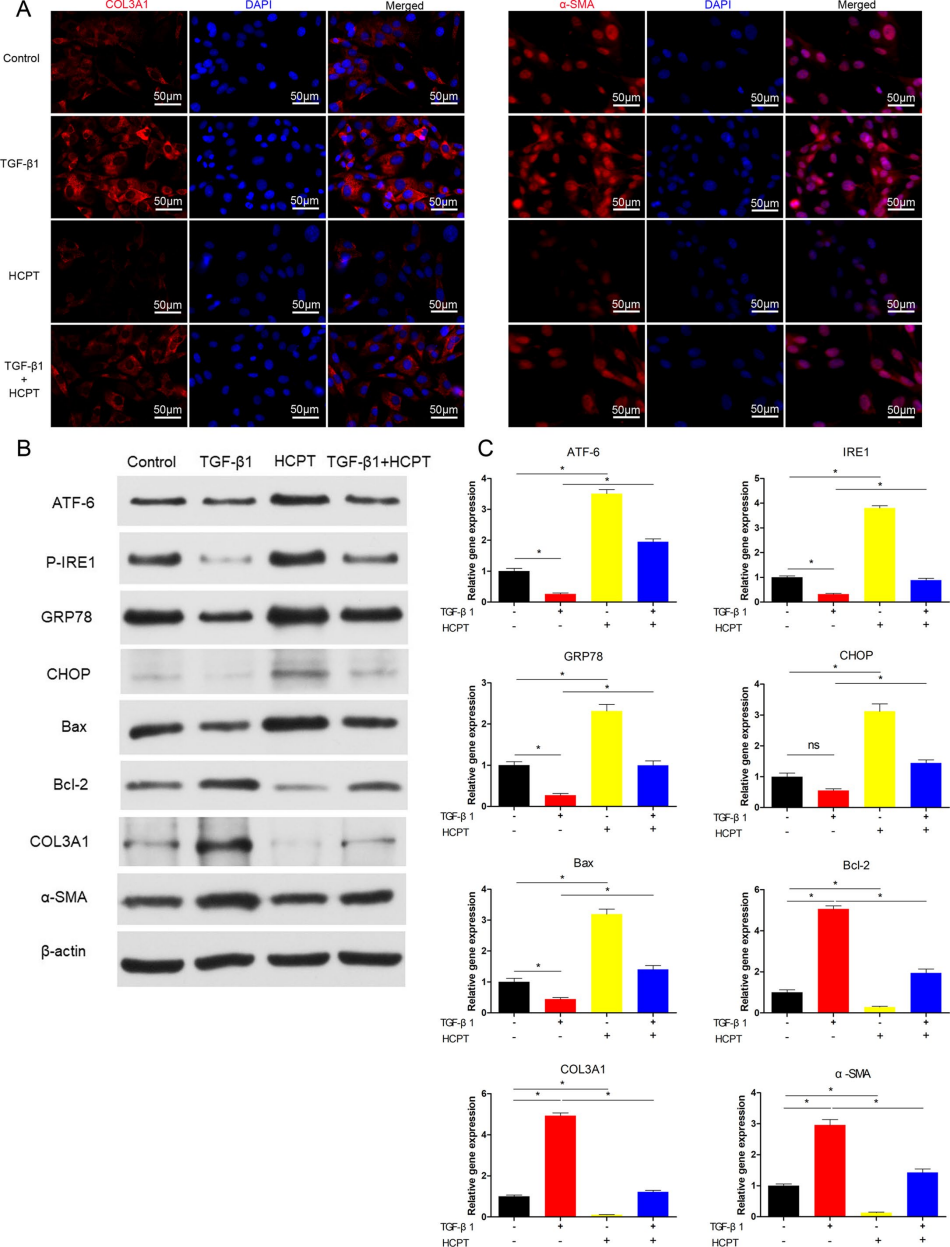
HCPT inhibited TGF-β1-induced adhesion *in vitro*. Fibroblasts were incubated with HCPT and/or TGF-β1 for 24 h. **(A)** Representative cellular immunofluorescence pictures of COL3A1 and α-SMA. **(B)** Representative western blot pictures of ATF-6, P-IRE1, GRP78, CHOP, Bax, Bal-2, COL3A1, and α-SMA. **(C)** Relative mRNA expression levels of ATF-6, IRE1, GRP78, CHOP, Bax, Bal-2, COL3A1, and α-SMA. Data are showed as the mean ± SD of five independent samples. *P < 0.05. ns: not significant.

We further evaluated the effects of HCPT on fibrosis *in vitro*. IF was applied to analyze the expression level of α-SMA and COL3A1. The results show that the treatment of fibroblasts with 2 ng/ml TGF-β1 for 24 h significantly increases the expression of COL3A1 and α-SMA, and this increase is significantly inhibited by the addition of 1 μg/ml HCPT (**Figure 4A**). Western blot and q-PCR results further confirmed the inhibitory effect of HCPT on ECM deposition (**Figures 4B, C**). From the above findings, we found that HCPT suppresses TGF-β1-induced peritendinous adhesion *in vitro*.

### HCPT Activates ER Stress *in Vitro*

Our previous studies showed that HCPT could activate the PERK pathway *in vitro*. In the present study, we further investigated the effects of HCPT on other ER stress pathways. Fibroblasts were treated with TGF-β1 for 24 h, and western blot results revealed that ER stress proteins such as ATF-6, P-IRE1, GRP78 and CHOP were significantly decreased; moreover, the expression of Bcl-2 increased and the proportion of Bax/Bcl-2 decreased. However, HCPT treatment resulted in opposing trends. In addition, HCPT significantly reversed the changes in the TGF-β1-induced protein levels (**Figure 4B**). The results of q-PCR further confirms the activation of ER stress-related genes by HCPT (**Figure 4C**). These findings suggest that HCPT may trigger the ER stress pathway to promote fibroblast apoptosis *in vitro*.

### HCPT Inhibits Tendon Fibrosis by Activating IRE1

**Figure 5:**
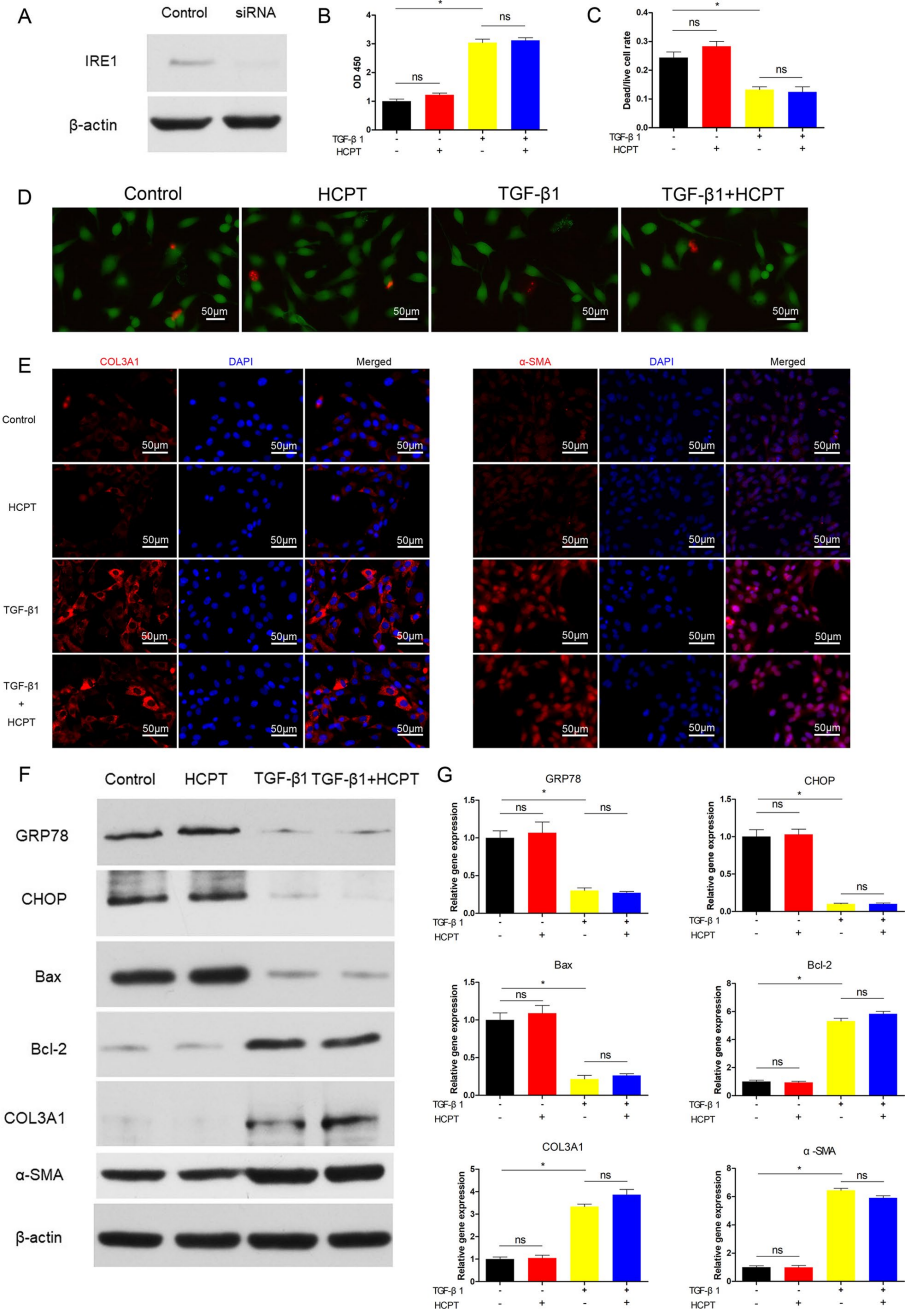
SiRNA targeting IRE1 abolished the inhibitory effect of HCPT on TGF-β1-induced fibroblast fibrosis. Fibroblasts were pretreated with siRNA targeting IRE1 and then incubated with TGF-β1 and/or HCPT for 24 h. **(A)** Representative western blot images of IRE1. **(B)** The CCK-8 assay of cell viability. **(D)** Representative pictures of dead/live staining and **(C)** dead/live rate analysis. **(E)** Representative cellular immunofluorescence pictures of COL3A1 and α-SMA. **(F)** Representative western blot pictures of GRP78, CHOP, Bax, Bal-2, COL3A1, and α-SMA. **(G)** Relative mRNA levels of GRP78, CHOP, Bax, Bal-2, COL3A1, and α-SMA. Data are showed as the mean ± SD of five independent samples. *P < 0.05. ns: not significant.

To further clarify the relevant mechanisms between HCPT and the ER stress pathways, we determined whether the inhibition of fibrosis by HCPT was IRE1 dependent. Prior to TGF-β1 and/or HCPT treatment, siRNA targeting IRE1 was applied to pre-treated fibroblasts according to the manufacturer’s instructions. The western blot results showed that the expression of IRE1 was significantly reduced (**Figure 5A**). We examined fibroblast proliferation and apoptosis after IRE1 knockdown. The CCK-8 results indicated that the effect of HCPT on cell proliferation was eliminated (**Figure 5B**). The dead/live rate analysis further revealed that the effect of HCPT-induced fibroblast apoptosis was abolished after IRE1 transfection pretreatment (**Figures 5C, D**). In a further study, we investigated the inhibitory effect of HCPT on fibrous characterization after IRE1 knockdown in fibroblasts. The western blot and q-PCR results indicated that HCPT did not improve the TGF-β1-induced changes in the expression of COL3A1 and α-SMA (**Figures 5F, G**). A similar conclusion was also reached in the IF analysis (**Figure 5E**). Furthermore, HCPT did not reverse the TGF-β1-induced alterations in genes involved in the ER stress pathway (**Figures 5F, G**). These data suggest that HCPT suppresses TGF-β1-triggered peritendinous fibrosis by activating the IRE1 signaling pathway.

### HCPT Inhibits Tendon Fibrosis by Activating ATF-6

**Figure 6:**
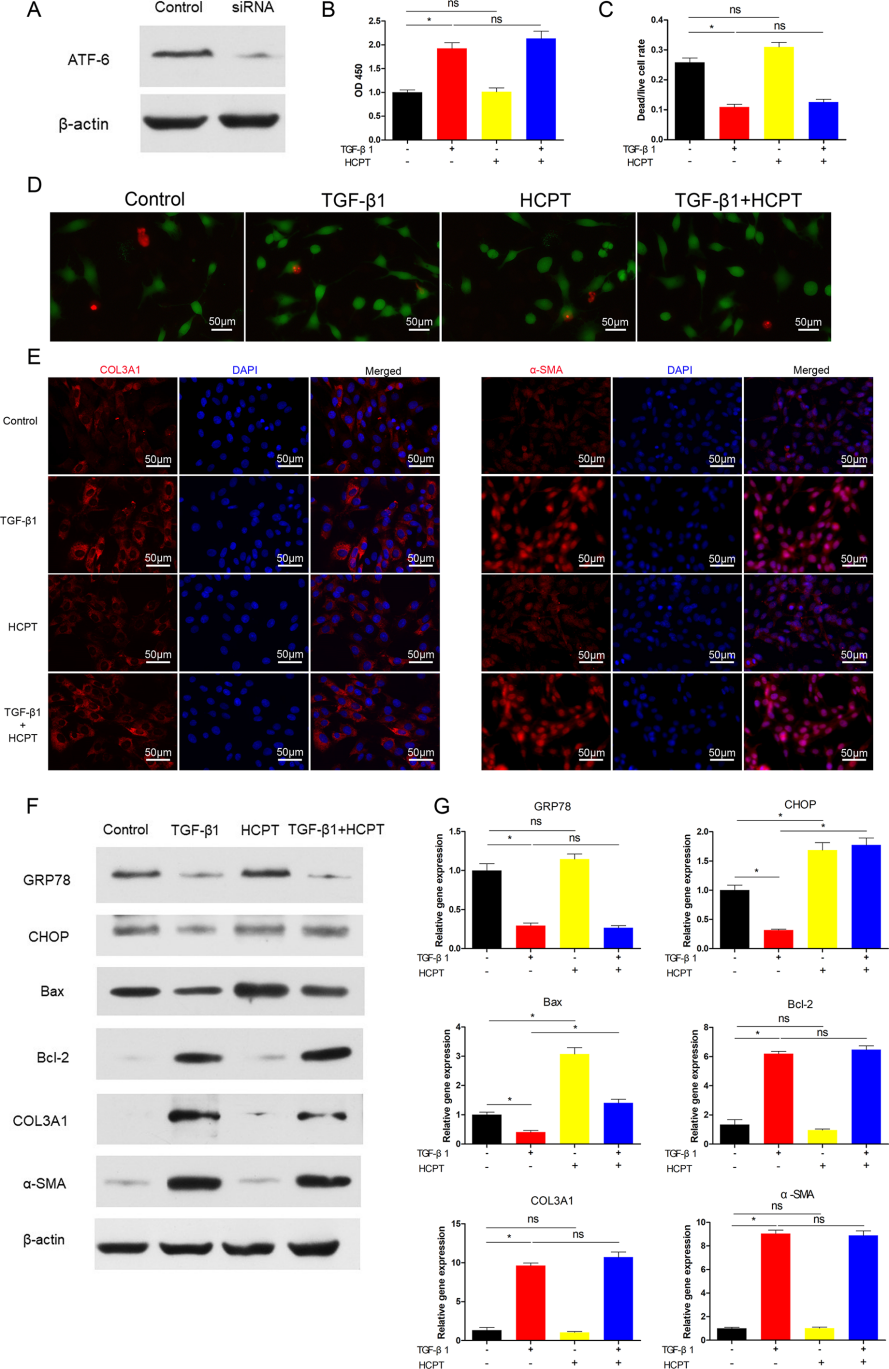
SiRNA targeting ATF-6 blocked the inhibitory effect of HCPT on TGF-β1-induced fibroblast fibrosis. Fibroblasts were pretreated with siRNA targeting ATF-6 then incubated with TGF-β1 and/or HCPT for 24 h. **(A)** Representative western blot image of ATF-6. **(B)** The CCK-8 assay of cell viability. **(D)** Representative pictures of dead/live staining and **(C)** dead/live rate analysis. **(E)** Representative cellular immunofluorescence pictures of COL3A1 and α-SMA. **(F)** Representative western blot pictures of GRP78, CHOP, Bax, Bal-2, COL3A1, and α-SMA. **(G)** Relative mRNA expression levels of GRP78, CHOP, Bax, Bal-2, COL3A1, and α-SMA. Data are showed as the mean ± SD of five independent samples. *P < 0.05. ns: not significant.

**Figure 7:**
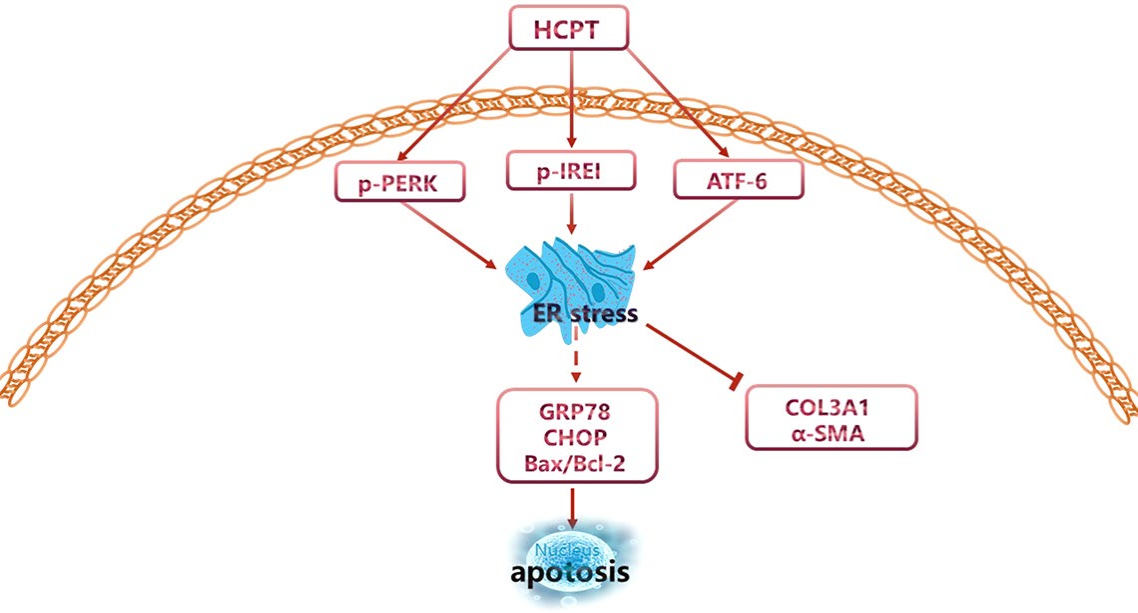
Potential signaling pathways involved in the regulation of peritendinous adhesion by HCPT.

Next, we determined whether the inhibition of fibrosis by HCPT was ATF-6-dependent. Similar to the IRE1 experiment, siRNA targeting ATF-6 was applied to pre-treated fibroblasts followed by incubation with TGFβ1 and/or HCPT. The western blot results revealed a remarkable decline in the expression of ATF-6 (**Figure 6A**). CCK-8 and dead/live rate analyses showed that the apoptosis phenomenon induced by HCPT in fibroblasts disappeared after ATF-6 knockdown treatment (**Figures 6B–D**). The western blot, q-PCR, and IF analyses showed that HCPT did not inhibit fibrosis following knockdown of ATF-6 (**Figures 6E–G**). Regarding ER stress-related genes, HCPT failed to reverse the TGF-β1-induced changes in GRP78 and Bcl-2, whereas the effects of HCPT on CHOP and Bax remained following the knockdown of ATF-6. Both the western blot and q-PCR results confirmed this conclusion (**Figures 6F, G**). These data indicates that the ATF-6 partial pathway is activated and involved in the inhibition of fibrosis by HCPT. Based on our previous research (Yao et al., 2019) and the above findings, the potential pathway by which HCPT inhibits tendon fibrosis is presented in **Figure 7**.

## Discussion

In this study, we found that HCPT relieved peritendinous adhesion after tendon injury. First, we observed that topical application of HCPT relieved tendon adhesion in a concentration-dependent manner in a rat model of tendon injury, in which the decrease in ECM deposition was paralleled by the activation of the ER stress pathways. Second, we confirmed in *in vitro* experiments that HCPT promoted fibroblasts apoptosis and suppressed the secretion of ECM. Importantly, the inhibitory effect of HCPT on tendon fibrosis was effectively blocked by siRNAs targeting ER stress genes ATF-6 and IRE1, providing a clue to the mechanism underlying the role of HCPT in tendon adhesion.

The pathological features of fibrotic lesions include inflammation, myofibroblast transformation, excessive ECM deposition and tissue dysfunction (Wynn and Ramalingam, 2012). Peritendinous fibrosis, which is a common complication, has similar pathological features, mostly in cases of tendon injury, tendon surgery and arthrodesis. As an antitumor drug with few side effects, HCPT regulates the cell cycle and induces apoptosis, suggesting its potential beneficial effects in fibrotic diseases. Studies in recent years have increasingly confirmed this beneficial effect (Sun et al., 2008; Yang et al., 2011; Yin et al., 2013).

Our previous studies demonstrated that HCPT regulates proliferation and apoptosis in fibroblast and thus has the potential to prevent tendon adhesion (Yao et al., 2019). In this study, using a rat tendon injury model, we observed that 0.1 mg/ml HCPT significantly inhibited peritendinous adhesion 3 weeks after injury. It is worth noting that sometimes prevention of peritendinous fibrosis may be counterproductive to tendon healing and repair (Jiang et al., 2014). In this study, no significant difference was found in the maximum tensile strength of the rat tendon with or without HCPT treatment, indicating that tendon healing is not affected. Next, TGF-β1 was employed to mimic the pathological features of fibroblast adhesion *in vitro*. The data shows that HCPT effectively suppresses TGF-β1-triggered cell proliferation and promotes apoptosis while reducing ECM secretion. These results are consistent with the phenomena observed *in vivo*.

The ER stress is regulated by the activation of the ER resident proteins PERK, IRE1, and ATF-6 for the purpose of restoring ER homeostasis. Otherwise, ER stress will result in programmed cell death (Wu and Kaufman, 2006). Our previous study showed that HCPT could activate the PERK pathway in human fibroblasts to induce apoptosis. However, it was unclear whether HCPT acts on the other two branches of ER stress when preventing tendon adhesion. In this study, it was found that IRE1 and ATF-6 in the ER stress pathway and downstream molecules is activated by HCPT, paralleling HCPT-induced fibroblast apoptosis and reduced ECM secretion. This finding has been confirmed in both *in vivo* and *in vitro* experiments.

To further confirm the relationship between HCPT and the ER stress pathway, we employed siRNAs targeting IRE1 and ATF-6 to the explore possible mechanisms. The results show that the knockdown of IRE1 and ATF-6 blocked the ability of HCPT to inhibit TGF-β1-induced tendon fibrosis. Notably, after knockdown of ATF-6, the effect of HCPT on CHOP and Bax remained. In contrast, after IRE1 knockdown, the effects of HCPT on the ER stress pathway were all abolished. A possible explanation for these results may be that ER-dependent apoptosis is mediated not only by CHOP, but also by the activation of caspase 12 and caspase 2. Caspase 2 activates the pro-apoptotic protein BID, which activates Bax and Bak (Guo et al., 2002; Courreges et al., 2019). Unfortunately, our study did not involve the effects of silencing ATF-6 or IRE1 on each other. It has been reported that ATF-6 activation, in addition to helping cells to process accumulated unfolded proteins, also induces X box binding protein 1 (XBP1) gene (Adachi et al., 2008), which is critical for cell survival. IRE1 signaling is currently thought to be at the core of regulating ER stress-induced apoptosis (Jäger et al., 2012). Prolonged IRE1 signaling may protect against PERK-mediated apoptosis by XBP1 splicing (Lin et al., 2007; Lin et al., 2009). This seems to imply some degree of synergy between IRE1 and ATF-6, however, few related studies have been reported.

Recently, Mo et al. revealed that after knockdown of IRE1 or XBP1, TGF-β1/TM-induced Epithelial-mesenchymal transition was blocked in the progression of pulmonary fibrosis (Mo et al., 2015). This finding is consistent with our results. Furthermore, Courreges et al. found that ANP stimulated ER-dependent programmed cell death to alleviate acute pancreatitis, during which the expression of ATF-6 was elevated in the presence of ANP (Courreges et al., 2019). In our experiments, it was found that HCPT induced an increase in the expression of ATF-6, and the effect of inhibitory fibrosis of HCPT disappeared after knockdown of ATF-6. These findings corroborate our conclusion that HCPT inhibited TGF-β1-induced peritendinous fibrosis *via* IRE1- and ATF-6-dependent programmed cell death. In addition, we previously reported that HCPT induced fibroblast apoptosis *via* the PERK pathway. Collectively, we cautiously concluded that HCPT inhibited peritendinous adhesion through the ER-dependent apoptotic pathway. Although there have been studies on HCPT inhibition of fibrotic diseases, we demonstrated for the first time the potential of HCPT as an anti- peritendinous drug. Second, we systematically demonstrated that HCPT inhibited tendon adhesion by regulating ER stress-mediated fibroblasts apoptosis. However, this study had limitations. The effect of HCPT on tenocytes was not investigated *in vitro*. The dose of HCPT used *in vivo* has not been optimized and requires further research.

In conclusion, our study provides evidence that HCPT is a promising avenue for the possible treatment and prevention of peritendinous adhesion. ER-dependent cell death may be a key mechanism of action and may become a new target for the treatment of tendon adhesion.

## Data Availability

The datasets generated for this study are available on request to the corresponding author.

## Ethics Statement

The animal study was reviewed and approved by Male Sprague-Dawley (SD) rats were purchased from the Shanghai Laboratory Animal Company (Shanghai, China) and were fed in specific pathogen-free (SPF) environment. Animal welfare was offered to all the experimental animals approved by the Animal Care Committee of Shanghai Jiao Tong University Affiliated Sixth People’s Hospital (No:2019-0239), and all procedures were conducted in accordance with standard guidelines.

## Author Contributions

ZY: designed the study, performed the experiments, and drafted the manuscript. WW: performed the experiments and analyzed the data. JN: collected and analyzed the data. XZ: analyzed the data. WZ contributed new reagents and analytic tools. YQ: interpreted data and approved the final version of manuscript. CF: designed the research.

## Funding

The study was supported by the National Natural Science Foundation of China (No. 81672146) and the Projects of Science and Technology Development Foundation of Pudong New District, Shanghai, China (Grant Nos. PKJ2016-Y55 and PWZxq2017-03).

## Conflict of Interest Statement

The authors declare that the research was conducted in the absence of any commercial or financial relationships that could be construed as a potential conflict of interest.

## Abbreviations

α-SMA, α-smooth muscle actin; ATF-6, activating transcription factor 6; CHOP, C/EBP homologous protein; ECM, extracellular matrix; ER, endoplasmic reticulum; GRP78, glucose regulated protein 78; HCPT, hydroxycamptothecin; IRE1, inositol requiring kinase 1; PERK, RNA-dependent protein kinase-like ER; siRNA, small interfering RNA;TGF-β1, transforming growth factor-β1.
